# Supplementary material for: Predicting Kidney Transplantation Outcomes from Donor and Recipient Characteristics at Time Zero: Development of a Mobile Application for Nephrologists
Source: J Clin Med. 2024 Feb 23;13(5):1270. doi: 10.3390/jcm13051270 (PMC10932177; doi:10.3390/jcm13051270)

Online Supplementary Material

Figure S1. Kidney Transplant App: Graft and patient survival models

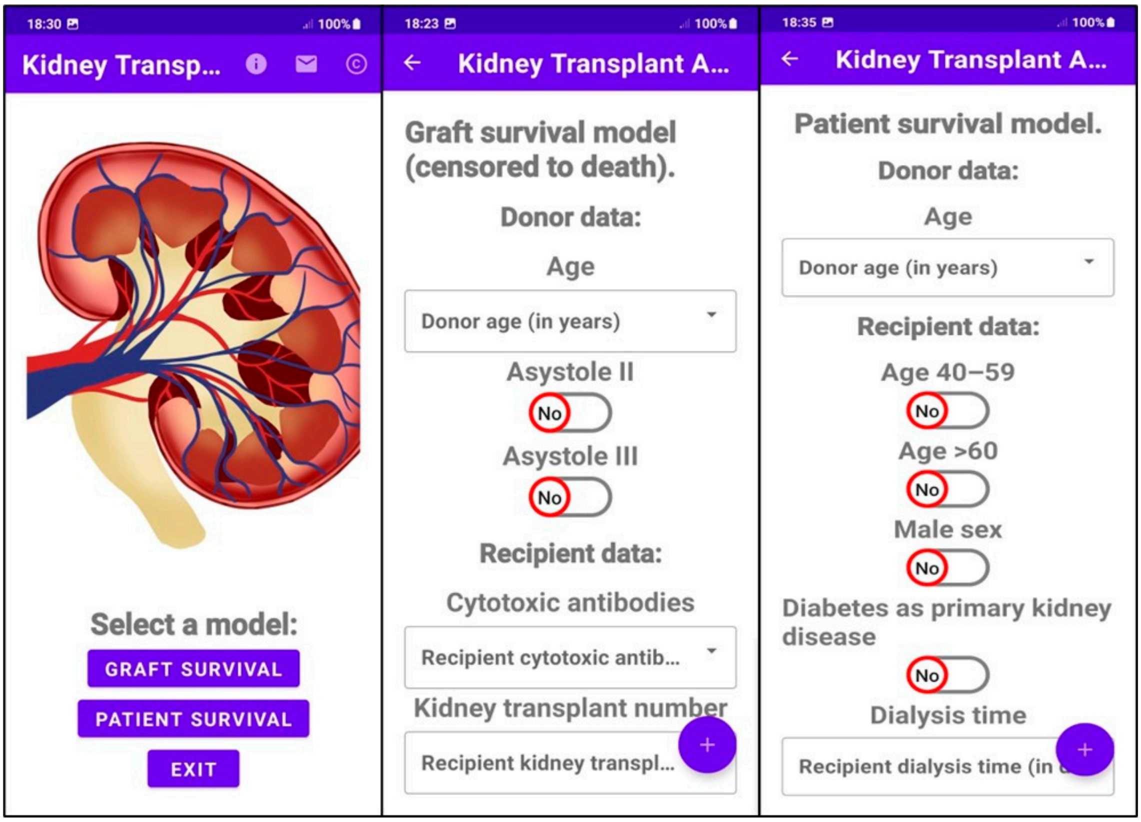

Figure S2. Kidney Transplant App: Example of graft survival calculation

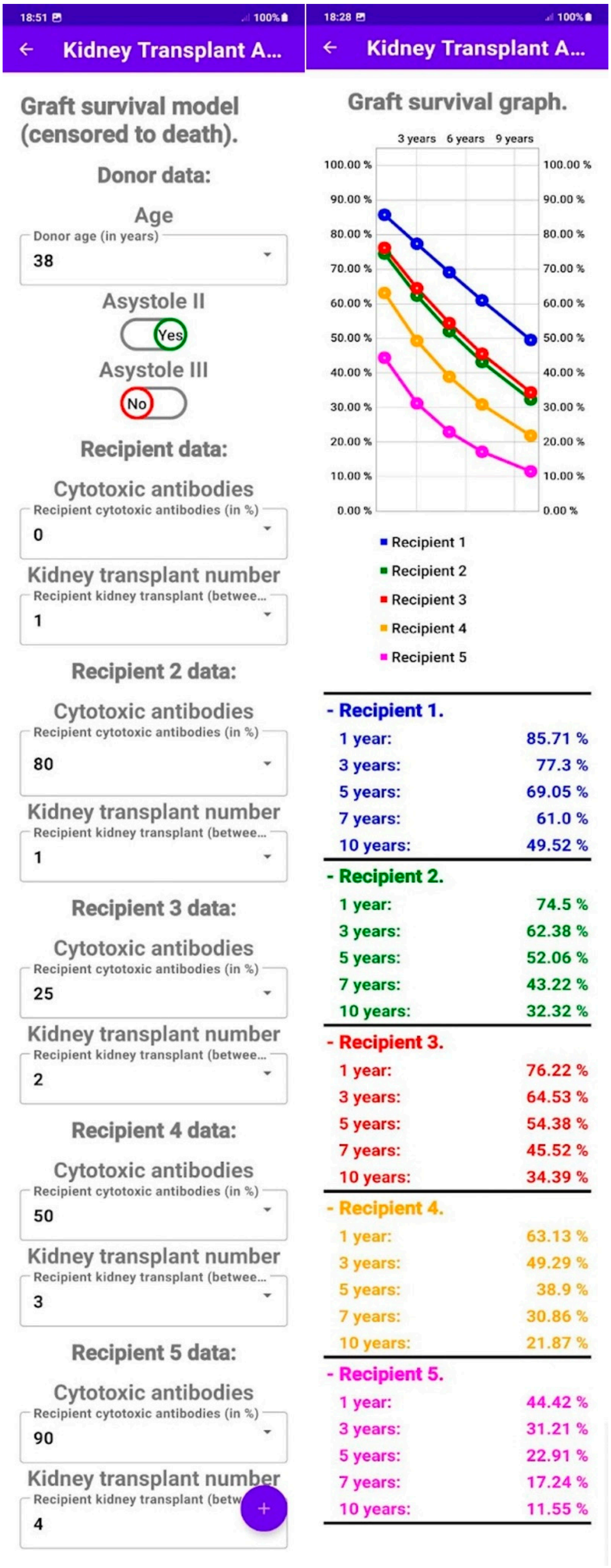

Figure S3. Kidney Transplant App: Example of patient survival calculation

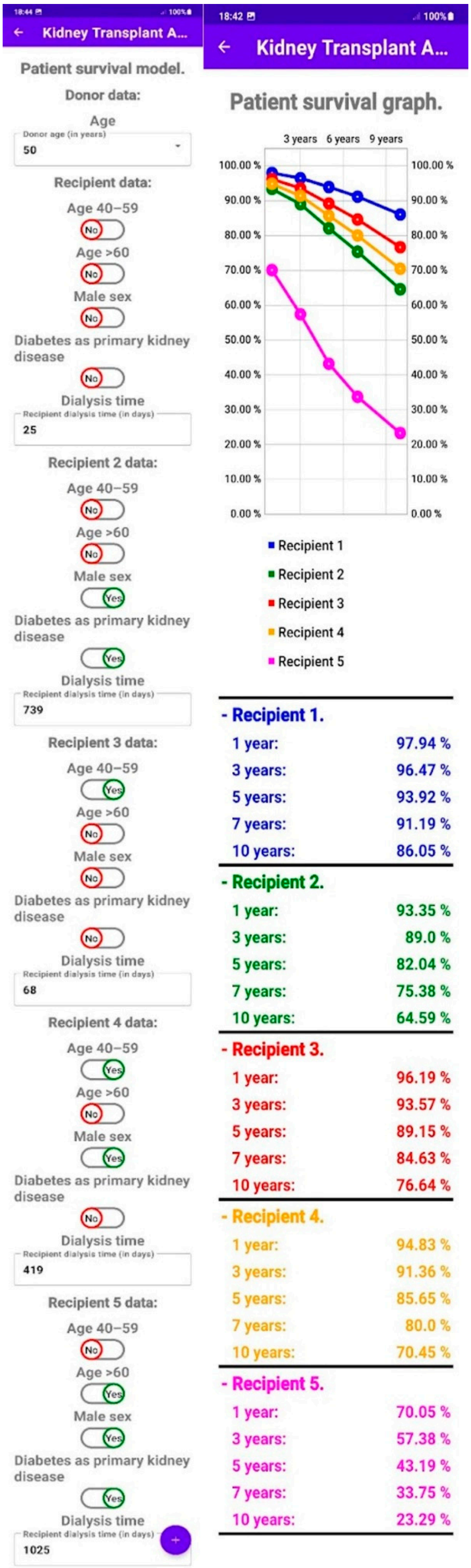

Supplement: Supplementary file 1 [file jcm-13-01270-s001.zip › Online Supplementary Material.pdf]
